# Reproductive aspects of the poorly known and critically endangered freshwater snail *Heleobia atacamensis* (Gastropoda: Truncatelloidea)

**DOI:** 10.7717/peerj.11550

**Published:** 2021-08-17

**Authors:** Gonzalo A. Collado, Elizabeth Chihuailaf, Natalia Muñoz-Herrera, Manuel Contreras, Fernando Novoa, Moisés A. Valladares

**Affiliations:** 1 Departamento de Ciencias Básicas, Facultad de Ciencias, Universidad del Bío-Bío, Chillán, Chile; 2 Centro de Ecología Aplicada, Santiago, Chile

**Keywords:** Development type, Protoconch, Reproductive cycle, Sex ratio, Sexual dimorphism, Shell shape variation

## Abstract

Knowing the reproductive biology of threatened species is essential for conservation and to establish proper management plans. *Heleobia atacamensis*, a freshwater snail only known from two locations in the Atacama Saltpan, northern Chile, is currently classified as Data Deficient on the IUCN Red List and Critically Endangered by the Ministerio del Medio Ambiente of Chile. Based on size-frequency distribution, multivariate analysis of shell measurements, and microdissections, we studied the reproductive strategy, recruitment period, sex ratio and sexual dimorphism in this species. *Heleobia atacamensis* is an oviparous species, with direct development (non-planktotrophic). Females lay capsules of a single egg from which a juvenile resembling a miniature adult hatches after intracapsular metamorphosis is completed. The development type was confirmed by the observation of a paucispiral protoconch (= protoconch I) using scanning electron microscopy. Recruitment was observed across the four seasons of the year, with an increment at the end of austral summer. Results also showed that sex ratio was 1:1, whereas sexual dimorphism was not detected using univariate and multivariate analysis of the shell. The reproductive data provided in this study are a starting point for future management plans.

## Introduction

Mollusca constitute the most important and largest phylum of invertebrate animals after Arthropoda. Estimations of mollusk species number range from 70,000–76,000 (Rosenberg, 2014) to 120,000 (Ponder, Hutchings & Chapman, 2002). It is not surprising that as a result of this high diversity, in addition to colonizing terrestrial, marine and freshwater habitats, they also display a variety of reproductive strategies. The group includes hermaphroditic, dioecious and parthenogenetic species. Sexual reproduction can occur either by internal or external fertilization; development types include oviparous species, with direct and indirect development, as well as ovoviviparous species (Purchon, 1977; Jakubik, 2006; Neves, Valentin & Figueiredo, 2010; Collado et al., 2019) whereas there may also be semelparous and iteroparous species, which may or may not present sexual dimorphism.

Extinction of biodiversity is currently occurring at an alarming rate because of habitat destruction, pollution, overhunting, poaching, fishing, pesticides and invasive species (Lowe, Browne & Boudlejas, 2000; Pimm & Raven, 2000; Beketov et al., 2013; Ceballos, Ehrlich & Dirzo, 2017; Comizzoli & Holt, 2019). Knowing the reproductive biology of overexploited or threatened species is essential for the maintenance of populations and implement management plans (Holt, Brown & Comizzoli, 2014). However, reproduction is sufficiently known in only a few species. Comizzoli & Holt (2019) have suggested that more basic and applied research in reproductive biology is required to preserve wild species and that such studies should be addressed considering different approaches. Basic research is aimed at understanding sexuality (hermaphroditic *vs*. gonochoric species), life cycle, reproductive strategy (fertilization type, oviposition pattern, development type), sexual maturity, fecundity, recruitment period, litter size, sexual differentiation between males and females, sex ratio and reproductive behavior, among other factors (Shine, 1989; Abouheif & Fairbairn, 1997; Kenchington & Glass, 1998; Gaillard, Coulson & Festa-Bianchet, 2008; Burks, Kyle & Trawick, 2010; Prida, Valenzuela & Astorga, 2018; Manlik, 2019).

*Heleobia* Stimpson, 1865 is the most diverse genus of the family Cochliopidae Tryon, 1866, including nearly 100 species (Hershler & Thompson, 1992; Cazzaniga, 2011). However, despite this high diversity there is little information about the reproductive biology of species of the genus (Szarowska et al., 2014). It has been reported in South America that adult females deposit individual gelatinous capsules during the reproductive season, each with an egg inside, which are attached to the surface of the shell of adult conspecifics or other substrates such as plants and stones (Marcus & Marcus, 1963, 1965; Cazzaniga, 1982; Martin, 2008; Collado & Méndez, 2011; Collado, 2015; Collado et al., 2020). To date, the reproductive cycle has been investigated in four species of *Heleobia*, which correspond to different lineages (Kroll et al., 2012; Collado, Valladares & Méndez, 2013; Koch, Martin & Ciocco, 2015; Collado, Valladares & Méndez, 2016): *Heleobia australis* (d’Orbigny, 1835), *Heleobia parchappii* (d’Orbigny, 1835), *Heleobia conexa* (Gaillard, 1974) and *Heleobia piscium* (d’Orbigny, 1835), for which two methods have been used: size-frequency distribution (Cazzaniga, 1981; Cazzaniga, 1982; De Francesco & Isla, 2004a; De Francesco & Isla, 2004b; Martin, 2008; Carcedo & Fiori, 2012; Merlo, Parietti & Etchegoin, 2016) and gonadal histology (Cazzaniga, 1982; Martin & Díaz, 2016).

Evaporitic saline lakes are ecosystems with a unique diversity that currently is being threatened in many cases due to climate change and human activities (Williams, 2002; Wurtsbaugh et al., 2017). One of these systems is the Atacama Saltpan in northern Chile, which has a high degree of endemism (Philippi, 1860; Jerez, 2000; Castro et al., 2006). The freshwater snail *Heleobia atacamensis* (Philippi, 1860) is a poorly known species endemic to this system. It was described from Tilopozo in the southern margin of the saltpan but subsequently recorded from Peine, north of Tilopozo (Collado, Valladares & Méndez, 2013). Apart from distribution and occurrences, available data for *H. atacamensis* only refer to penis morphology, indicating a gonochoric sexuality (Collado et al., 2011; Collado, Valladares & Méndez, 2013). The species was assessed as Data Deficient by the IUCN Red List (Pastorino & Darrigan, 2011) and as Critically Endangered (CR) according to Rules of Classification of Wild Species (Reglamento de Clasificación de Especies Silvestres in Spanish) of the Ministerio del Medio Ambiente de Chile (RCE DS MMA), considering the extreme endemism and declining habitat quality (MMA, 2014).

The aims of this study were to determine the reproductive strategy of *H. atacamensis* regarding the oviposition pattern and development type, to assess sexual dimorphism and sex ratio, and to determine recruitment periods of the species, essential elements for eventual management and conservation.

## Materials & Methods

Snails were randomly collected from Tilopozo, Atacama Saltpan, northern Chile (23°46′34.73″S; 68°14′9.83″W), using a hand sieve and stored in 70% ethanol. The authors were authorized by the Undersecretary of Fisheries, Republic of Chile, to obtain the biological material in four season sampling (Exempt Resolutions No. 1253/2018 and 1720/2019). Sampling was performed in November, 2018 (*n* = 145), and March (*n* = 141), May (*n* = 65) and July, 2019 (*n* = 165).

To determine the oviposition pattern and development type, we performed stereomicroscope observations of shell surface of the adults to find egg capsules attached (Cazzaniga, 1982; Collado & Méndez, 2011; Collado, 2015; Collado et al., 2020) or in the rest of the fixed sample, including newly hatched juveniles. The development type was also studied through the observation of the individual larval shell (= protoconch) found on the apex of adult shells (= teloconch) using scanning electron microscopy. Planktotrophic species, that is, those with indirect development, develop two types of larval shells: protoconch I, which appears during early development (intracapsular); and protoconch II, which appears during the more advanced free-swimming phase (Bouchet, 1990). This type of protoconch is also called multispiral protoconch; it consists of 2–5 whorls, and is generally sculptured. The species with direct development (non-planktotrophic) develop only the protoconch I, also called paucispiral protoconch, which is simpler, consisting of 1–2 whorls, generally unsculptured (Bouchet, 1990). We isolated the apex of adult shells containing the protoconch and placed them in a diluted hypochlorite solution (1:3) for 10–15 min to remove material attached (mainly sediment and diatoms). The protoconch was observed using a scanning electron microscope (SEM) Hitachi SU3500.

To study recruitment period, size-class structure and sexual dimorphism, we photographed and measured the shell of each individual using a Motic SMZ-168 stereomicroscope equipped with a Moticam 2000 digital camera. To define recruitment period and prevailing size classes, the shell length (SL) was measured using a millimeter ruler to construct seasonal size-frequency distributions considering all the animals collected. To assess sex ratio, we performed microdissections in 74 individuals, which were sexed as male if they had a penis in the head, behind the right tentacle, and as female if they lacked this feature. For this we used individuals whose SL was greater than 2.5 mm, considering that Cazzaniga (1982) determined in *H. parchappii* reproductive adult and subadult individuals of a size greater than or equal to this value. The snails were also considered appropriate for this type of study since they had 4 or 5 spire whorls. The numbers of males and females were compared using the chi-square test assuming an expected ratio of 1:1 for sexually reproducing species (Hamilton, 1967). To assess sexual dimorphism in external shell morphology, we compared the SL and four other shell variables (shell width: SW, aperture length: AL, aperture width: AW, body whorl length: BW) using the non-parametric Mann–Whitney *U* test (*U*-test; *p* > 0.05) in STATISTICA v. 7.0 (StatSoft Inc., 2004) since some of variables were not normally distributed. A principal component analysis (PCA) was implemented to visualize the variation present in shell shape of males and females and evaluate the occurrence of distinct groups in the morphospace. All variables (Table S1) were log transformed for these analyses.

## Results

### Oviposition pattern and development type

**Figure 1:**
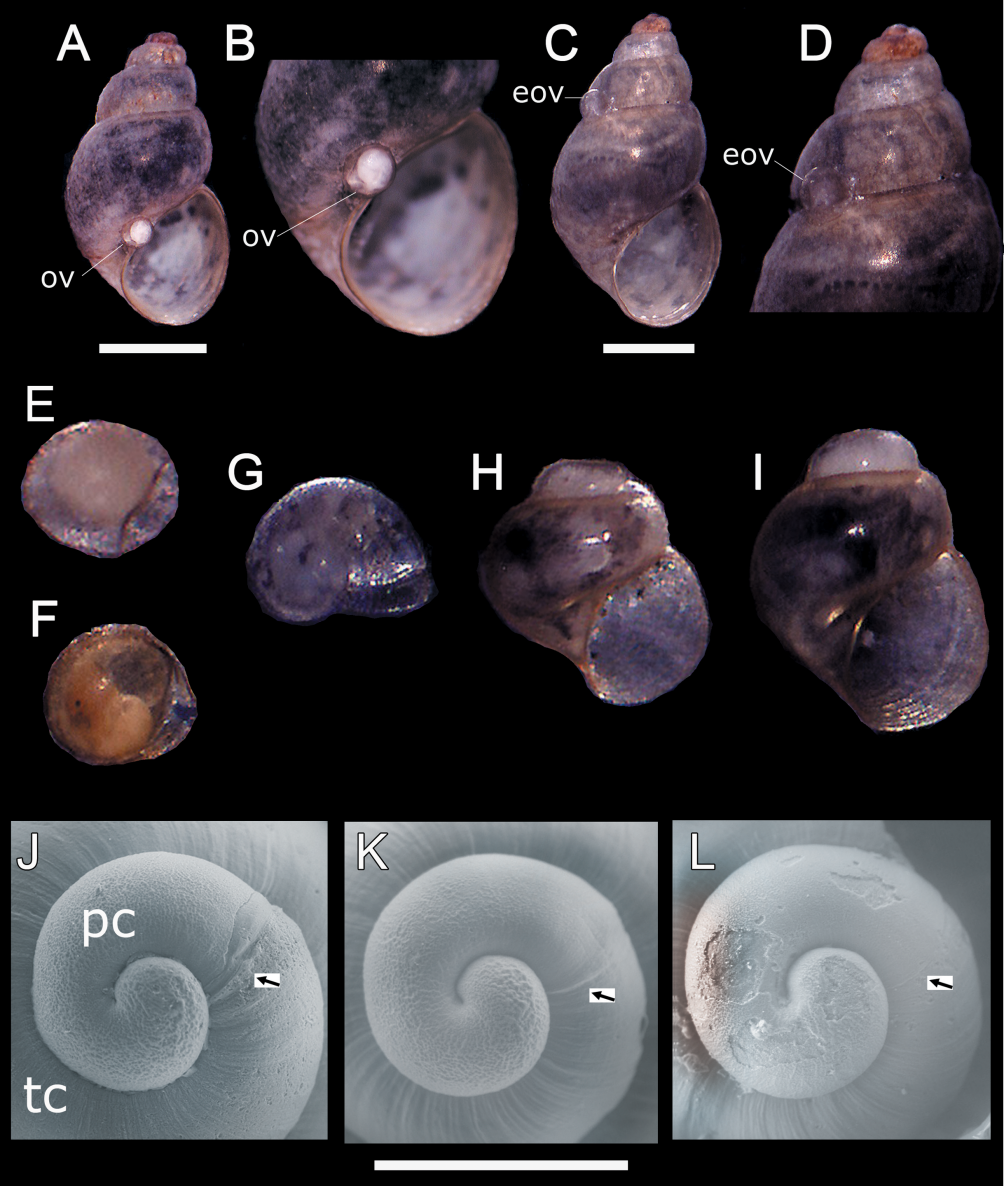
Oviposition pattern and protoconch of *Heleobia atacamensis*. (A) Shell of an adult having an ovicapsule adhered to the edge of the peristome with a shell-bearing juvenile inside. (B) Same specimen seen at higher magnification. (C) A second adult with an empty ovicapsule (hatched) attached to the shell surface. (D). Same specimen seen at higher magnification. (E) Unhatched ovicapsule with a shell-bearing juvenile inside (330 μm). (F) Unhatched ovicapsule with a shell-bearing juvenile inside (330 μm) in a later stage of development than the juvenile shown in E. (G) A newly hatched juvenile represented by the protoconch and an incipient teloconch (500 μm). (H, I) Two juveniles (1.0 and 1.2 mm, respectively) with protoconch and teloconch. (J–L) Protococh of different specimens (the arrow indicates the boundary of the protoconch with the teloconch). eov, empty ovicapsule; ov, ovicapsule; pc, protoconch; tc, teloconch. Scale: A, C = 1 mm; J–L = 300 µm.

In all sampling months we found single egg capsules (= ovicapsule) containing a shell-bearing juvenile inside attached to the shell surface of adult individuals (Figs. 1A–1F). We also found newly hatched juveniles in all the samples, which resemble miniature adults (Fig. 1G), and juveniles a little larger than those recently hatched (Figs. 1H and 1I). Based on SEM observations, *H*. *atacamensis* presents only protoconch I (Figs. 1J–1L), of the paucispiral type. This larval shell generally has ridges surrounding small depressions in its initial part, and smooth at the end. With a length of 331.7 ± 12.1 μm (*n* = 6), this shell reaches somewhat less than 1 whorl. The border between the proto- and teloconch is visible in *H*. *atacamensis*, although some apices were eroded.

### Sex ratio

Of the 74 sexed snails, 39 were males and 35 females. Male SL ranged from 2.6 to 5.0 mm and females from 2.5 to 4.7 mm. Although the number of males was greater than females, the sex ratio did not differ significantly from 1:1 (X^2^ = 0.21, *p* > 0.05).

### Sexual dimorphism

**Figure 2:**
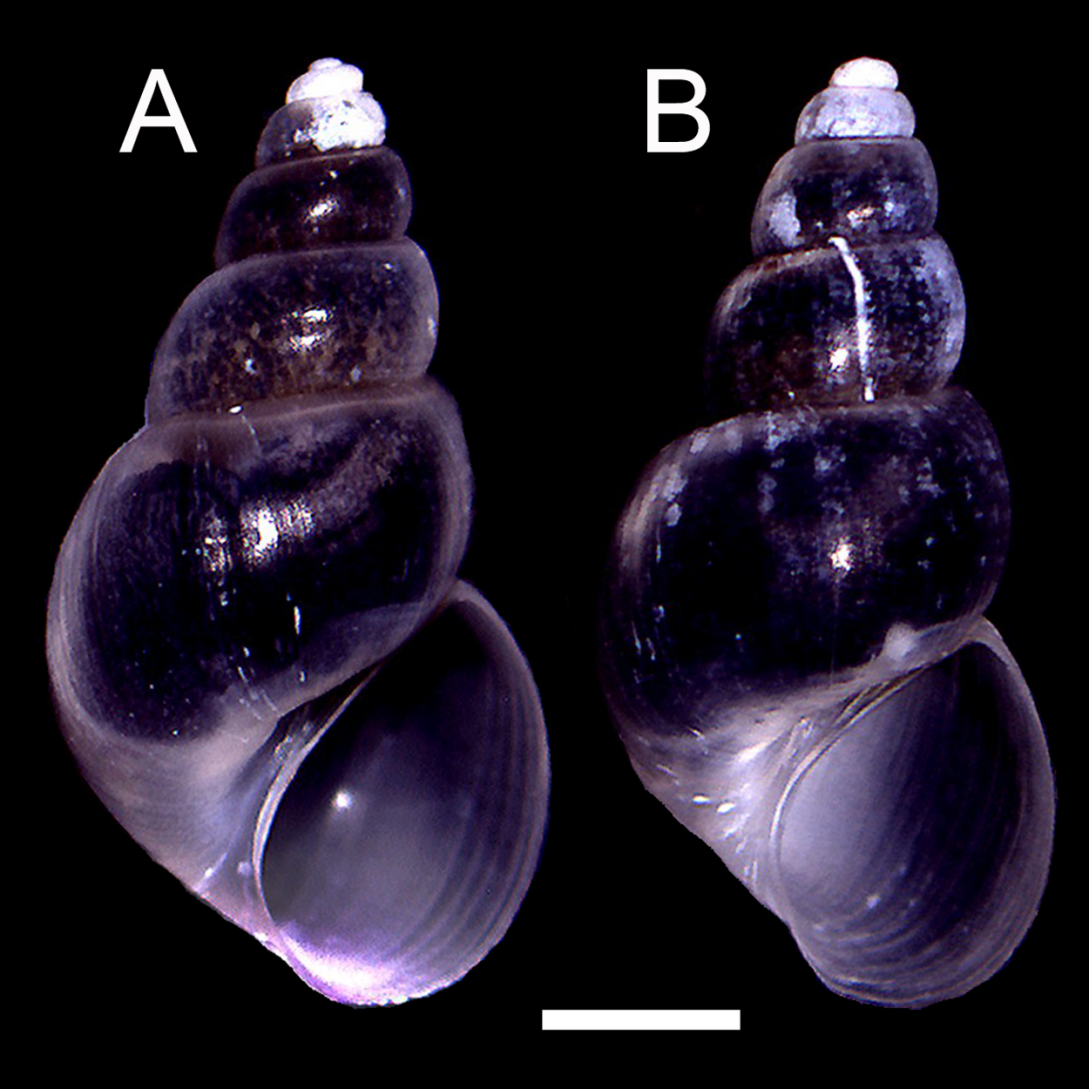
Adult shells of *Heleobia atacamensis*. (A) Female, (B) male. Scale: 1 mm.

**Figure 3:**
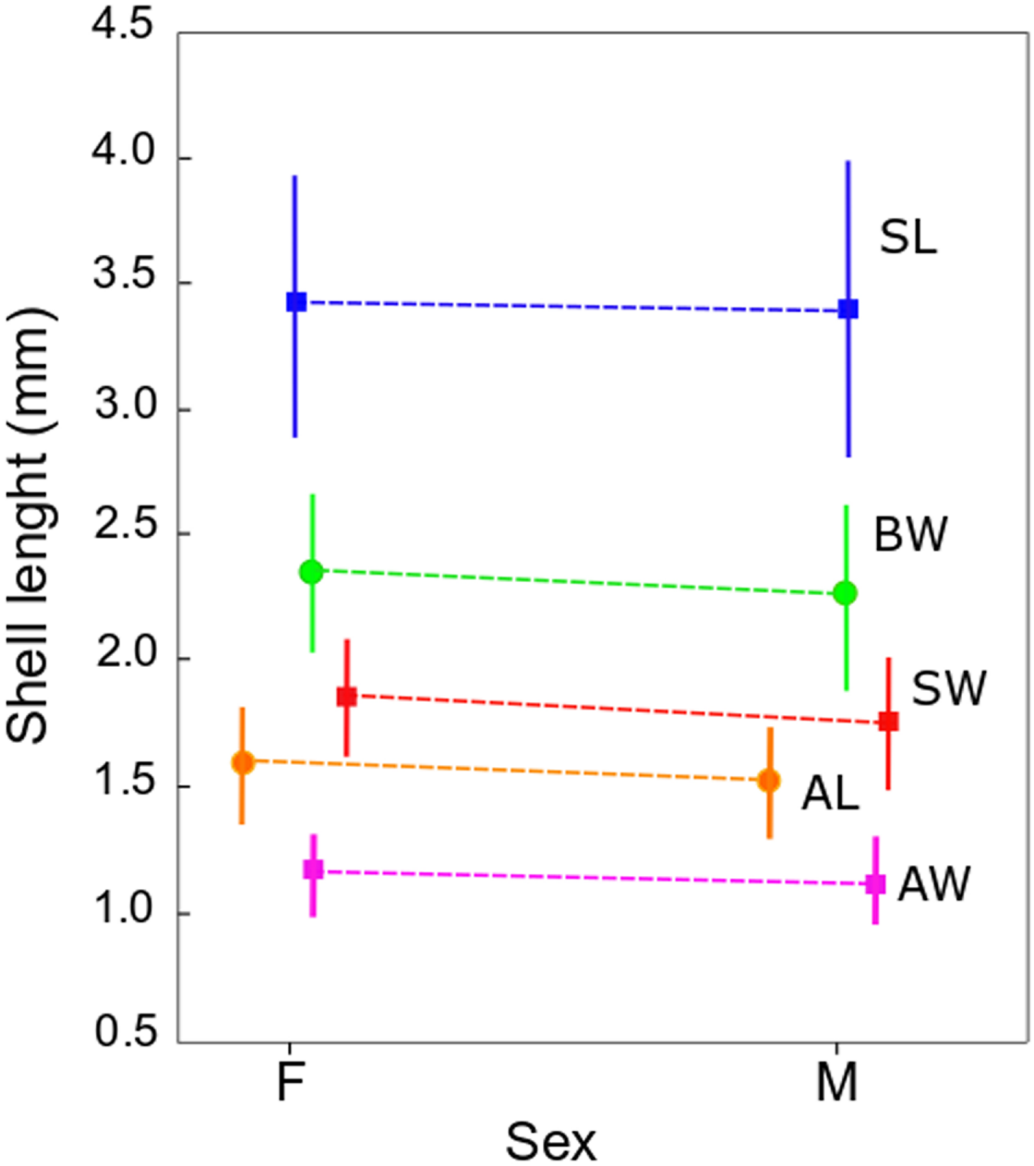
Shell measurements in *Heleobia atacamensis*. Plot of means of the five shell variables comparing females (F) and males (M). Vertical bars indicate standard deviation. SL: shell length, SW: shell width, AL: aperture length, AW: aperture width, BW: body whorl length.

**Figure 4:**
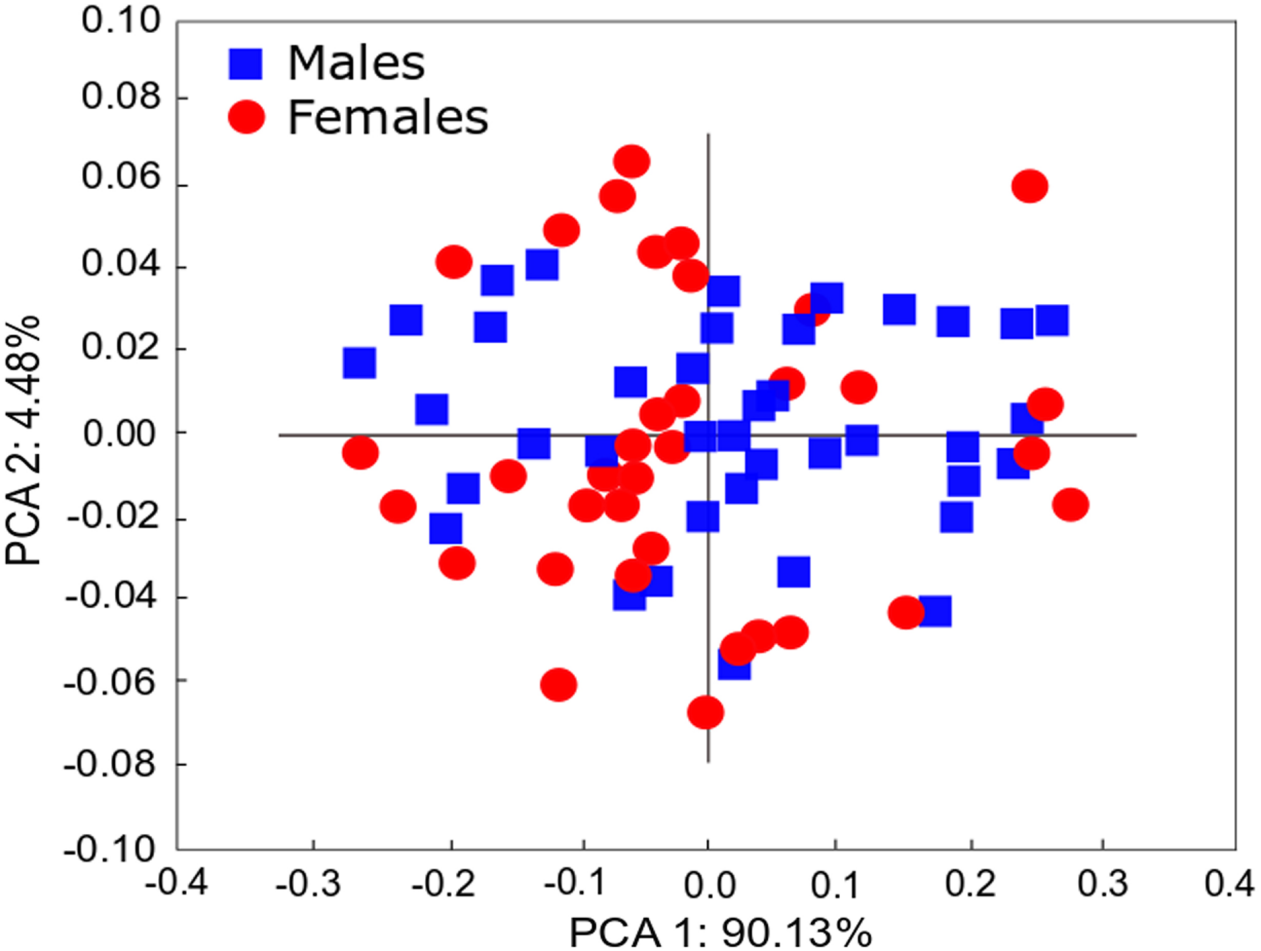
PCA of *Heleobia atacamensis*. Scatter plot comparing females and males. ****

The qualitative observation of the external morphology of the shell did not differentiate females from males in *H. atacamensis* (Fig. 2). The sculpture and color of the shell and protoconch did not distinguish between the sexes. The univariate analysis of shell variables showed non-significant differences between sexes (*U*-test, *p* > 0.05) (Table 1) (Fig. 3). The first component of the PCA explained 90.13% of the total variance in the data, while the second one 4.48% (Fig. 4). The clusters of points, representing males and females, overlap strongly, with several individuals of both sexes grouping very closely in the morphospace.

**Table 1:** Shell comparison (Mann–Whitney *U* test) between male and females of *Heleobia atacamensis*.

Shell variable	Mean (± sd) ♀	Mean (± sd) ♂	*U*	*p*-level
SL	3.4 ± 0.5	3.4 ± 0.6	620.5	0.504304
SW	1.9 ± 0.2	1.8 ± 0.3	516.0	0.072163
AL	1.6 ± 0.2	1.5 ± 0.2	542.5	0.130151
AW	1.2 ± 0.2	1.1 ± 0.2	588.5	0.310785
BW	2.4 ± 0.3	2.3 ± 0.4	555.5	0.170122

**Note:**

The differences were not statistically significant (p > 0.05).

### Recruitment period

**Figure 5:**
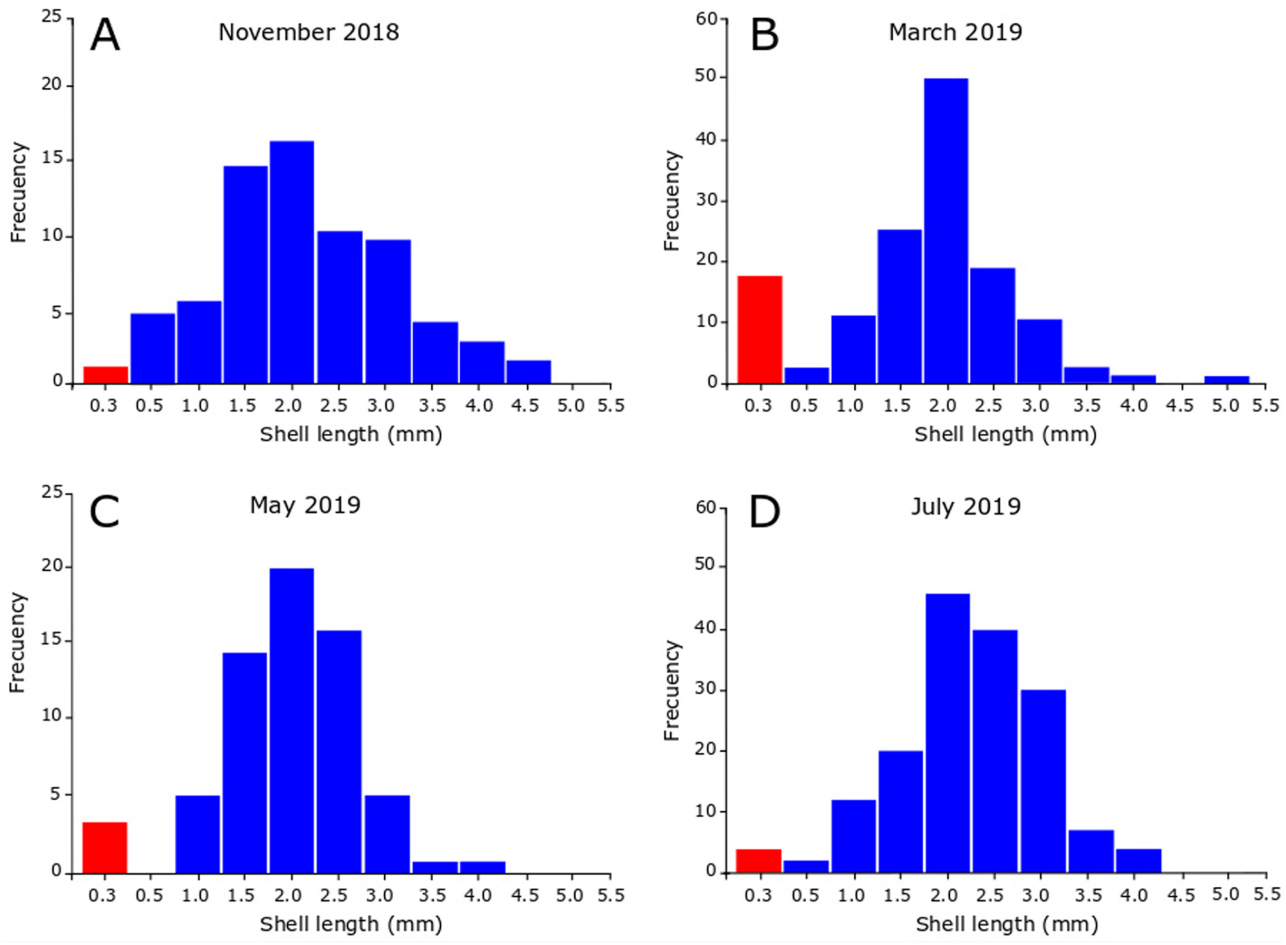
Seasonal size-frequency distributions of *Heleobia atacamensis* from Tilopozo, Atacama Saltpan. (A) November 2018, (B) March 2019, (C) May 2019, (D) July 2019. Red bars indicate the size class of individuals within ovicapsules or recently hatched (recruits).

In November 2018, the snails varied between 0.35 and 4.90 mm in SL, revealing the presence of juveniles and adults in the sample (Fig. 5A). Figure 5A also shows that the highest number of juveniles (shell length less than 2.5 mm) were grouped in class marks 1.5 mm (14 individuals) and 2 mm (16 individuals). The sample contained unhatched ovicapsules with shell-bearing juveniles inside (2 individuals). In March 2019 (Fig. 5B) the SL of snails varied between 0.40 and 5.40 mm. Small-sized juveniles were also found, but in this case in greater numbers than November 2018. The sample contained two unhatched ovicapsules with shell-bearing juveniles inside. In May 2019 (Fig. 5C), the individuals varied in size between 0.35 and 4.10 mm. Two unhatched ovicapsules with shell-bearing juveniles inside were also found. In July 2019 (Fig. 5D), the SL varied between 0.45 and 4.30 mm. Two newly hatched shell-bearing juveniles from ovicapsules were found. Figures 5B–5D also shows that the highest number of juveniles were grouped in class marks 1.5 and 2 in March (25 and 50 individuals, respectively), May (14 and 20 individuals, respectively) and July (20 and 44 individuals, respectively).

## Discussion

Knowing the type of development is essential for the conservation of a species. In the present study we showed that *H. atacamensis* is an oviparous species with direct development, according to the presence of ovicapsules adhered to the adult shells containing a shell-bearing juvenile inside and the finding of newly hatched juveniles at the same time. We also demonstrated that one of the oviposition sites is the shell of conspecifics and that newborns hatch in the same habitat of the adults. The presence of a paucispiral protoconch (protoconch I) is also indicative of direct development (Bouchet, 1990). For production of juvenile snails of *H. atacamensis* in culture, direct development lacks the difficulties inherent in a free-swimming phase (=planktonic larval stage), for example, foraging or predation. Other species of *Heleobia* such as *Heleobia chimbaensis* (Biese, 1944), *Heleobia deserticola* Collado, 2015, *Heleobia peralensis* Collado et al., 2020, *Heleobia miaulis* (Marcus & Marcus, 1965) and *H. parchappii* also have direct development (Marcus & Marcus, 1965; Cazzaniga, 1982; Collado et al., 2011; Collado, 2015; Collado et al., 2020). *Heleobia charruana* (d’Orbigny, 1840) and *H. australis* have indirect development. This last species attaches single egg capsules to living shells and opercula of conspecifics from which a free-swimming veliger larva hatches 5 days post-oviposition (Marcus & Marcus, 1963). The veliger has a simple bilobed velum, suggesting a short pelagic larval life (Neves, Valentin & Figueiredo, 2010). In *H. charruana*, free-swimming larvae hatch 10 days post-oviposition, remaining among the aquatic plants (Marcus & Marcus, 1963) by which they can be dispersed by rafting; that would not be the case of *H. atacamensis*.

Adult sex ratio has implications on fitness, breeding potential, behavior, mating system, sexual selection, life history, population demography, invasiveness of alien species and conservation biology (Le Galliard et al., 2005; Skalski, Ryding & Millspaugh, 2005; Kokko & Jennions, 2008; Liker et al., 2015; Székely, Weissing & Komdeur, 2014; Pipoly et al., 2015; Xu et al., 2016; Carmona-Isunza et al., 2017; Rodriguero et al., 2019). The sex ratio in natural populations may vary due to genetic causes, pollution and environmental factors, and may even present temporary differences (Hirai et al., 2004; Yusa, 2007; Ciparis, Henley & Voshell, 2012; Neves, Valentin & Figueiredo, 2013; Carmona-Isunza et al., 2017). In this study we found that the sex ratio in *H. atacamensis* from Tilopozo was 1:1. A population with a sex ratio biased to one of the sexes has been reported to have a higher probability of extinction (Courchamp, Clutton-Brock & Grenfell, 1999). Although there may be stochastic variations in the sex ratio, a 1:1 value implies that the population of *H. atacamensis* from Tilopozo is in equilibrium (Hamilton, 1967). If there is an eventual culture in this locality (in situ conservation) or transplantation of individuals from Tilopozo to other natural systems or captivity (*ex situ* conservation), a sample of individuals from this locality will surely include both males and females. Xu et al. (2016) reported for the golden apple snail *Pomacea canaliculata* (Lamarck, 1828) that the sex ratio is influenced by temperature and precipitation, while Yusa (2007) linked the variation to genetic causes. Ciparis, Henley & Voshell (2012) also found that population sex ratios in snails of the genus *Leptoxis* Rafinesque, 1819 vary according to the water temperature. Neves, Valentin & Figueiredo (2013) mentioned that the sex ratio in *H. australis* can be affected by organotin compound, mainly tributyltin (TBT). It is unknown if any environmental variable affects the sex ratio in *H. atacamensis*.

Sexual dimorphism in animals, a pattern in which males and females of the same species differ in some trait, is a common feature among many gonochoric taxa; it is exhibited in a variety of ways (Berns, 2013; Páll-Gergely et al., 2020). However, in Caenogastropoda, a very large and diverse group which includes *H. atacamensis*, external sexual dimorphism is commonly absent (Páll-Gergely et al., 2020). In the present study, the analysis of shell variables did not reveal statistically significant differences between males and females. The study also did not reveal qualitative differences in the shape or surface of the shell, and the PCA showed overlap between individuals of both sexes. The impossibility of detecting males and females would constitute a problem when selecting specimens for potential management of the species. Nevertheless, for *in situ* or *ex situ* conservation, minute truncatelloid snails can be sexed by keeping them alive in Petri dishes so that they can be turned over using tweezers with the shell opening pointing upwards; in a few seconds the penis protrudes from the opening of the shell on the neck of the males (G.A. Collado, 2017, personal observations; see also Smith, 2012). Andreeva et al. (2017) showed absence of sexual dimorphism in *Parafossarulus manchouricus* (Gerstfeldt in Bourguignat, 1860), a species morphologically similar to *H. atacamensis*. Schilthuizen et al. (2003) did not detect sexual dimorphism in external shell morphology in *Plectostoma concinnum* (Fulton, 1901) (*s.l*.) using shell measurements, but the sexes can be differentiated by the shell color, including the protoconch (Schilthuizen, Sipman & Zwaan, 2017). Jordaens, Platts & Backeljau (2001) found that females of *Pomatias elegans* (Müller, 1774) are slightly larger than males, but such difference is not sufficient to discriminate between sexes. Páll-Gergely et al. (2020) found a strong sexual dimorphism in *Barnaia longituba* (Páll-Gergely & Gargominy, 2017) and *Streptaulus blanfordi* (Benson, 1857), with females being substantially larger than males. Similarly, Reichenbach, Baur & Neubert (2012) reported for *Cochlostoma septemspirale* (Razoumowsky, 1789) that females are larger and wider than males. Such differences were not found in *H. atacamensis*.

It has been reported that the reproductive period in *Heleobia* species presents variations during the life cycle and may be influenced by biological and physical variables such as parasitism, predation, environmental characteristics and anthropogenic action (De Francesco & Isla, 2004a; De Francesco & Isla, 2004b; Carcedo & Fiori, 2012; Martin & Díaz, 2016; Merlo, Parietti & Etchegoin, 2016). The present study indicates that recruitment periods in *H. atacamensis* occur in all seasons of the year, mainly in the summer, probably influenced by the water thermal condition. It is important to note the presence of ovicapsules, newly hatched juveniles (recruits) and a large number of more developed juveniles in all sampling months. This suggests that *H. atacamensis* would reproduce continuously throughout the year. Knowing the reproductive season of any species is in itself important. However, this is essential in endangered species. In an eventual *in situ* or *ex situ* conservation effort for *H. atacamensis*, it would be possible to obtain reproducers of both sexes all year long, as well as juveniles, whose success would be increased in the summer months. As the species is restricted to the Atacama Saltpan, there are no other described populations of the species outside of this system that can contribute adults, juveniles or larvae for repopulation or culture.

The continuous reproductive pattern of *H. atacamensis* is consistent with data from other species of the genus. In the population of *H. parchappii* from Nahuel Rucá Lagoon, Argentina, recruitment occurs in the four seasons of the year (Merlo, Parietti & Etchegoin, 2016). In brackish waters of Mar Chiquita, Argentina, De Francesco & Isla (2004b) reported that this species reproduces from spring to autumn, being able to produce egg capsules throughout the entire sampling period (August 1998–August 1999). For the populations of *H. parchappii* from the Colorado River valley, Cazzaniga (1981) reported a high hatch rate in late spring and a smaller peak in winter. He also reported the absence of a gonadal recovery period, and suggested semelparity in this species. It is unknown if *H. atacamensis* is a semelparous (characterized by a single reproductive event before death) or iteroparous species (characterized by multiple reproductive cycles during its life cycle). In *H. australis* and *H*. *conexa* the reproductive activity occurs from spring (October) to early winter (June) in Mar Chiquita, being longer in *H. conexa* (De Francesco & Isla, 2004a). On the other hand, *H. australis* recruits once a year during the summer in the Bahía Blanca estuary, Argentina, with a long life cycle of approximately 30 months (~2.5 years). In this population, the recruits (<2.5 mm) represented a small percentage of all individuals (Carcedo & Fiori, 2012). Longer reproduction in *H. parchappii* and *H. conexa* could be associated with a more stable water condition of the particular lentic water bodies with respect to the more unstable estuarine environment of Bahía Blanca (De Francesco & Isla, 2004a; Alda et al., 2010). In the population of *H. piscium* from Martín García Island, Argentina, Martin (2008) reported that the reproductive season took place for 6 months according to size-frequency distribution estimates, extending from early summer to mid-autumn, judging by the presence of egg capsules adhered to the surface of the shell of some specimens. This pattern, based on a single reproductive effort, differs from *H. atacamensis* and other congeners studied. Using histology, Martin & Díaz (2016) showed that reproduction in *H. piscium* from Isla Martín García occurs from November (spring) to February (summer), without a resting period.

For poorly known species of minute size and threatened with extinction, it is important to know basic morphological attributes and reproductive features that can contribute to its identification and conservation (Collado & Fuentealba, 2020; present study). Breeding programs have been considered as an integral part of conservation plans for many critically endangered species (Jewgenow et al., 2017). *Heleobia atacamensis* is only known from Tilopozo and Peine in the Atacama Saltpan (Philippi, 1860; Collado, Valladares & Méndez, 2013), and therefore its classification as critically endangered in Chile is justified (MMA, 2014). However, although the data provided in this study are oriented to know reproductive aspects, the species remains poorly known since other ecological parameters (e.g. feeding, microhabitats, abundance) and other aspects of its life cycle (e.g. semel-iteroparity, fecundity, sexual maturity) have yet to be investigated. Moreover, it is not known what factors influence its reproduction. On the other hand, it is important to note that the Atacama Saltpan contains several isolated and semi-isolated freshwater ecosystems with undescribed populations of the genus that could possibly correspond to *H. atacamensis*, so determining the geographic distribution of the species is also crucial for purposes of conservation.

## Supplemental Information

10.7717/peerj.11550/supp-1 Supplemental Information 1 Shell variables (mm) used in this study to evaluate sex ratio and sexual dimorphism in *Heleobia atacamensis*. F: female; M: male. F: female; M: male.

## Additional Information and Declarations

### Field Study Permissions

The following information was supplied relating to field study approvals (i.e., approving body and any reference numbers): Field experiments were approved by the Undersecretary of Fisheries, Republic of Chile (Exempt Resolution No. 1253/2018 and 1720/2019).

### Data Availability

The following information was supplied regarding data availability: The raw measurements of sex ratio and sexual dimorphism are available in the Supplemental File.
